# Comparisons of clinical performance of guardian laryngeal mask with laryngeal mask airway ProSeal

**DOI:** 10.1186/s12871-015-0039-3

**Published:** 2015-05-01

**Authors:** Ajay Kumar Pajiyar, Zhiting Wen, Haiyun Wang, Lin Ma, Lumin Miao, Guolin Wang

**Affiliations:** 1 Department of Anesthesiology, Tianjin Medical University General Hospital, Tianjin Research Institute of Anesthesiology, No 154 Anshan Road, Heping District, Tianjin, 300052 China; 2 Department of Anesthesiology, Tianjin Children’s Hospital, Tianjin, 300074 China

**Keywords:** Guardian laryngeal mask airway, ProSeal laryngeal mask airway, Ambu cuff pressure gauge

## Abstract

**Background:**

The Guardian Laryngeal Mask Airway (G-LMA) is a new silicone-based single-use extraglottic device with the drainage port and a cuff pilot valve with pressure indicator. The aim of this study is to compare the clinical performance of this laryngeal mask airway with ProSeal laryngeal mask airway (P-LMA).

**Methods:**

In this prospective randomized study, we included adult patients with ASA grading I and II scheduled for elective surgery requiring supine position under total intravenous anesthesia. The patients were randomly allocated to two groups, 40 in each. G-LMA and P-LMA were used in groups G and P respectively. The cuff of each device was air inflated to 60 cmH_2_O. The primary outcome was to compare the airway sealing pressure and the secondary outcome was to compare the efficacy and safety of these two devices with respect to insertion success, insertion time, ease of insertion, volume of air for cuff inflation to 60 cmH_2_O, intracuff pressure measurement, gastric tube insertion attempt, gastric tube insertion time, Fiberoptic laryngeal view, and postoperative pharyngolaryngeal morbidity.

**Results:**

The airway sealing pressure at 60cmH_2_O cuff pressure was significantly greater in G-LMA than P-LMA (*p* = 0.04).The first successful attempt of both groups were comparable (*p* = 1.000). Insertion time was significantly shorter in G-LMA than P-LMA (*p* < 0.0001). The first successful attempt for the gastric tube insertion in both groups was comparable (*p* = 0.431). Gastric tube insertion time was less in G-LMA than in P-LMA (*p* < 0.0001). The volume of air for cuff inflation to 60 cmH_2_O was more in G-LMA than in P-LMA (<0.0001). The intracuff pressure measurement at 30, 60, 90 and 120 minutes were comparable (*p* = 0.823, 0.182, 0.870, 0.658).We did not find differences in ease of insertion (*p* = 0.60); Fiber-optic positions of airway devices were comparable (*p* = 0.83). In addition, blood staining (*p* = 1.00), sore throat and dysphagia at 1, 2 and 24 hour (*p* = 1.00) were comparable in both groups.

**Conclusion:**

The Guardian laryngeal mask airway was associated with high airway sealing pressure with a quicker insertion of the device as well as gastric tube.

**Trial registration:**

Clinical Trial.gov Identifier: NCT02063516. Date: June 2013

**Electronic supplementary material:**

The online version of this article (doi:10.1186/s12871-015-0039-3) contains supplementary material, which is available to authorized users.

## Background

**Figure 1:**
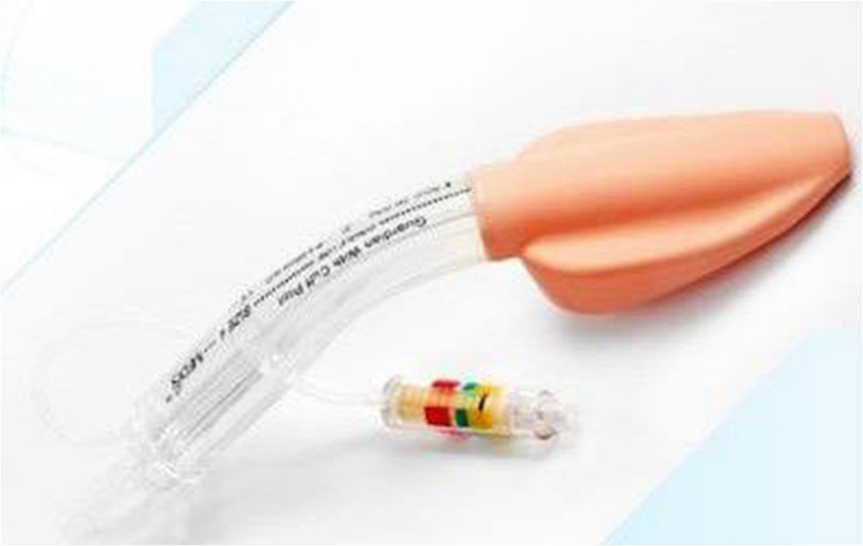
Guardian laryngeal mask airway with pilot balloon valve indicating pressure logo (Ultimate Medical Pty Ltd, Richmond, Vic, Australia).

The Guardian laryngeal mask airway (G-LMA) (Ultimate Medical Pty Ltd, Richmond, Vic, Australia) is a new silicone-based single–use extraglottic airway device that forms a seal with the glottis for ventilation, and with the hypopharynx for airway protection. It also provides a gastric drainage port (Figure 1). In addition, it has a port with suctioning material from the hypopharynx and a pilot balloon valve with pressure logo (Yellow <40 cmH_2_O, Green 40–60 cmH_2_O and Red >60 cmH_2_O) that indicate visual intracuff pressure (ICP) [1].This Laryngeal mask airway has been approved by SFDA (State Food and Drug Administration) for clinical application. The ProSeal laryngeal mask airway (P-LMA) (Laryngeal Mask Company, Henley-on-Thames, UK) is a reusable, silicone-based extraglottic airway device with a modified cuff to improve seal and a drain tube to provide channel for regurgitated fluid, prevention of gastric insufflation, and insertion of gastric tube [2,3]. We hypothesized that the differences in cuff design and airway tube results in differing effectiveness of glottic seal for ventilation. The aim of this study is to compare the clinical performance of G-LMA and P-LMA, and their postoperative pharyngolaryngeal morbidity. The primary outcome was to compare the airway sealing pressure (ASP) and the secondary outcome to compare the efficacy and safety of these two devices with respect to insertion success, insertion time, ease of insertion, volume of air for cuff inflation to 60 cmH_2_O, intracuff pressure (ICP) measurement, gastric tube insertion attempt, gastric tube insertion time, Fiberoptic laryngeal view, and postoperative pharyngolaryngeal morbidity.

## Methods

This study was approved by Tianjin Medical University General Hospital, Ethic Committee of Tianjin Medical University General Hospital, (Phone: +86-22-60361044). Approval number: IRB2013-035-01.

**Figure 2:**
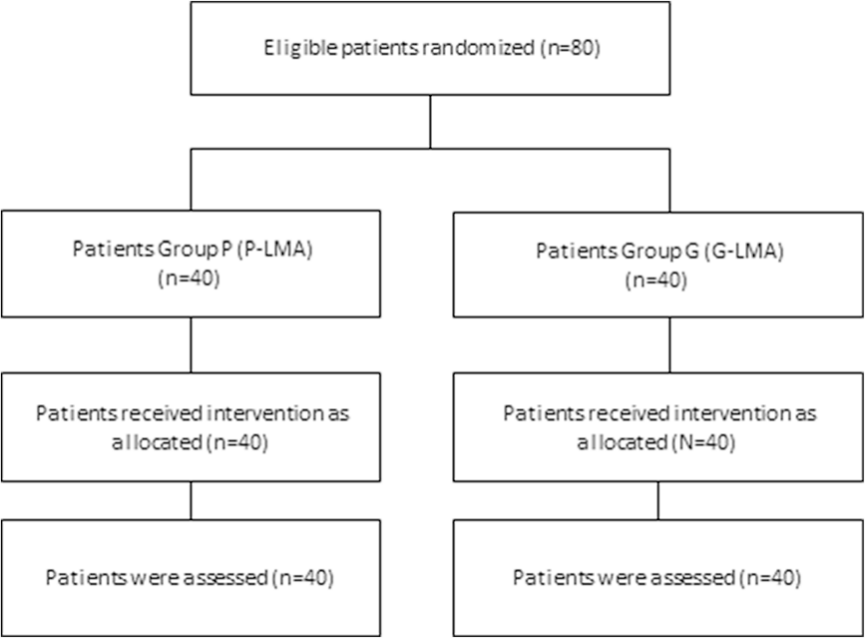
Patients flow chart (G-LMA vs. P-LMA).

It was conducted according to the guidelines of the above mentioned ethical requirements, and all patients gave written consent prior the study. 80 adult patients (ASA I and II) undergoing elective surgery in the supine position were randomly and prospectively enrolled in this study. The patients were randomly allocated to two groups - Group G where G-LMA was used and Group P where P-LMA was used; each group had 40 patients. The randomization was performed using sealed enveloped method from different surgical department (Figure 2). Patients aged less than 18 years, pregnant, with known difficult airway, body weight < 50 kg, body mass index (BMI) >35 kg/m2, cervical spine disease, incisor distance <2.5 cm, thyromental distance <6 cm, recent history of upper respiratory tract infection, history of gastro-esophageal reflux disease and full stomach were excluded from the study [4-6]. (Additional file 1).

Standard monitoring devices were attached before induction of anesthesia. After pre-oxygenation for 3 min, anesthesia was induced with intravenous injection of midazolam 0.05 mg/kg, sufentanil 0.4 μg/kg, and propofol 2–2.5 mg/kg. The rocuronium 0.6 mg/kg was given for neuromuscular blockade. The lungs were manually ventilated with oxygen via facemask for 3 min before the randomized airway device was picked up and inserted. Once the adequate depth of anesthesia was achieved, the assigned airway device was inserted. Only size 4 airway devices were used for adult patients weighing 50–70 kg. The insertion technique for both devices was identical to the recommended technique for laryngeal mask airway (LMA) and included head extension, neck flexion, full deflation of the cuff and a mid-line approach with index finger. Following insertion, the patients’ heads were stabilized in a neutral position. The cuff of airway device was air inflated to 60 cmH_2_O ICP and maintained throughout the surgery using cuff pressure gauge (Ambu Deutschland GmbH, Germany); the volume of air used was recorded. The number of insertion attempts was recorded. Two attempts were allowed before device use was considered a failure. Endotracheal intubation was performed when the randomized device failed. The insertion was scored as (1, insertion of device at first attempt without resistance; 2, insertion of device at first attempt with resistance; 3, insertion of device successful at second attempt; 4, insertion failed at second attempt) [7]. If a manipulation was required to achieve an effective airway, it was recorded as either ‘yes’ or ‘no’ and maneuvers required were recorded. The time between the picking up of the device and obtaining an effective airway was recorded. Effective ventilation was judged by bilateral symmetrical chest movement during manual ventilation, square wave on capnograph, no audible leak from the mouth and lack of gastric insufflation [8]. The device was fixed by taping the tube over the chin.

Once the effective airway was obtained, ICP was set at 60 cmH_2_O, and ASP determined by closing the expiratory valve of circle system at a fixed gas flow of 3 l/min. The airway pressure (maximum allowed, 40 cmH_2_O) at which an equilibrium was reached was recorded. The airway leak was detected by an audible sound of gas escaping from the mouth heard by listening closely to patient’s mouth [7]. ICP was measured at 30, 60, 90 and 120 minutes, and 60 cmH_2_O was maintained throughout the surgery.

A 14-French size gastric tube was used for both groups. A well-lubricated gastric tube was inserted into the stomach through gastric port of airway device. Correct placement was assessed by aspiration of gastric content or detection of injected air by epigastric auscultation [9].The time taken for correct placement was recorded (picking up the gastric tube until confirmation of placement). Failure was defined as inability to advance the gastric tube within two attempts. The anatomic position of airway devices was determined by passing flexible bronchoscope to a position just proximal to the end of the airway tube and scoring the view (1, Clear view of vocal cord; 2, Only arytenoids visible; 3, Only epiglottis visible; 4, No laryngeal structures visible) [3]. The patients’ lungs were ventilated with a tidal volume of 8 ml/kg, an inspiratory:expiratory ratio of 1:2, and the respiratory rate was adjusted according to an end-tidal CO_2_ of 35–40 mmHg with a fresh gas flow of 1.5 l/min. The anesthesia was maintained with continuous infusion of propofol 4–6 m/kg/h and remifentanil 0.1-0.3 μg/kg/min. A muscle relaxant was given in order to provide surgical relaxation. At the end of surgical procedure, anesthesia was discontinued. The airway device was removed when the patient was awake and obeyed verbal command. Following the removal of the device, it was checked for blood stained secretion and the observation was recorded. An independent staff member was solely responsible for recording postoperative pharyngolaryngeal morbidity (sore throat, dysphagia and dysphonia) at 1, 2 and 24 hours after surgery. The predetermined definition of postoperative pharyngolaryngeal morbidity for assessment: sore throat was defined as “constant pain or discomfort in the throat independent of swallowing”; dysphagia was defined as “difficulty or pain provoked by swallowing”; dysphonia was defined as “difficulty or pain on speaking”[10].

### Statistics

The primary outcome is the airway sealing pressure. The sample size was calculated to be 37 patients per group based on our study with the power of the test (1-beta) 80% and alpha level of 0.05 (for two-sided test). This gives an estimated difference of 2 cmH_2_O between groups, where (mean ± SD) of airway sealing pressure of GLMA was (32.36 ± 4.93 cmH_2_O) and PLMA was (29.41 ± 4.33 cmH_2_O) – as determined by an intracuff pressure of 60 cmH_2_O. In the case of any potential dropouts and guided by the above calculations, an optimal sample size of 40 patients in each group was chosen. The SPSS 17.0 statistical software system was used for statistical analysis. Device insertion time, volume of air for cuff inflation to 60 cmH_2_O, airway sealing pressure, gastric tube insertion time and ICP measurement were compared using Student *t*-test and Mann–Whitney test. Success rate of first insertion attempt, ease of insertion score, gastric tube insertion attempt, fiberoptic view and post-operative pharyngolaryngeal morbidity was compared using Chi-square test and Fisher exact test. A *P* value <0.05 was considered statistically significant.

## Results

The values are presented as mean ± SD or as numbers or as the number (%). There were no significant differences between the demographic profiles of patients and surgery types in the both groups [Table 1]. The results of airway sealing pressure, volume of air for cuff inflation to 60 cmH_2_O, ICP measurement, blood staining and postoperative pharyngolaryngeal morbidity are shown in Table 2. The results of successful insertion attempts, device insertion time, ease of insertion, successful gastric tube insertion attempt, gastric tube insertion time and fiberoptic position of airway devices are shown in Table 3.

**Table 1:** **Demographic characteristics and type of surgery performed**

Variables	Group P (P-LMA)(n = 40)	Group G (G-LMA)(n = 40)
**Age (years)**	45.20 ± 14.78	45.75 ± 13.58
**Sex (M/F)**	8/32	12/28
**Weight (kg)**	59.75 ± 4.72	61.65 ± 8.95
**Height (cm)**	160.30 ± 4.11	162.20 ± 4.86
**Body Mass Index (BMI) (kg/m** ^**2**^ **)**	23.27 ± 1.85	23.38 ± 2.71
**Type of surgery**		
**Gynecology**	20	18
**Orthopedics**	8	10
**Urology**	8	6
**General surgery**	4	6

P-LMA: ProSeal laryngeal mask airway; G-LMA: Guardian laryngeal mask airway.

**Table 2:** **Airway sealing pressure, cuff volume, intracuff pressure and pharyngolaryngeal morbidity**

Variables	Group P (P-LMA)(n = 40)	Group G (G-LMA)(n = 40)	*P*value
**Volume of air for cuff inflation to 60 cmH**_**2**_ **O, ml**	13.90 ± 1.19**	22.60 ± 3.27**	<0.0001**
**Airway sealing pressure, cmH**_**2**_ **O**	29.41 ± 4.33*	32.36 ± 4.93*	0.04*
**Intracuff pressure measurement, cmH**_**2**_ **O**			
**30 min**	58.83 ± 1.14	58.90 ± 1.15	0.823
**60 min**	58.67 ± 1.09	58.27 ± 1.20	0.182
**90 min**	58.40 ± 1.23	58.33 ± 1.35	0.870
**120 min**	58.27 ± 1.09	58.44 ± 1.03	0.658
**Blood staining on removal of device**	9/40 (22.5%)	8/40 (20%)	1.000
**Postoperative pharyngolaryngeal morbidity**			
**Sore throat**			
**1 h**	7/40 (17.5%)	6/40 (15%)	1.000
**2 h**	7/40	6/40	
**24 h**	0	0	
**Dysphagia**			
**1 h**	5/40 (12.5%)	4/40 (10%)	1.000
**2 h**	5/40	4/40	
**24 h**	0	0	
**Dysphonia**			
**1 h**	0	0	
**2 h**	0	0	
**24 h**	0	0	

**P < 0.01, *p < 0.05, P-LMA: ProSeal laryngeal mask airway; G-LMA: Guardian laryngeal mask airway; h: hours, min: minutes, ml: milliliter.

**Table 3:** **Device insertion characteristics, gastric tube insertion characteristics and fiber-optic view**

Variables	Group P (P-LMA)	Group G (G-LMA)	*P*value
	(n = 40)	(n = 40)	
**Successful insertion attempt**			
**First**	38 (95%)	39 (97.5%)	1.000
**Second**	2 (5%)	1 (2.5%)	
**Over all**	100%	100%	
**Device insertion time, seconds**	15.95 ± 1.84**	13.20 ± 1.82**	<0.0001**
**Ease of insertion**			
**1**	28	30	
**2**	10	9	
**3**	2	1	
**4**	0	0	
**Manipulation**	6/40	4/40	0.373
**Maneuvers**	6/40	4/40	0.373
**Successful gastric tube insertion attempt**			
**First**	35 (87.5%)	38 (95%)	0.431
**Second**	5 (12.5%)	2 (5%)	
**Overall**	40 (100%)	40 (100%)	
**Gastric tube insertion time, seconds**	13.75 ± 1.74**	10.05 ± 1.35**	<0.0001**
**Fiber-optic view**			
**(1/2/3/4)**	14/21/3/2	17/20/2/1	0.836

**P < 0.01, *p < 0.05, P-LMA: ProSeal laryngeal mask airway; G-LMA: Guardia laryngeal mask airway.

**Figure 3:**
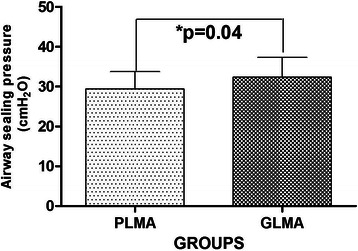
Comparison of airway sealing pressure in two groups (G-LMA vs. P-LMA). Results are given as means ± SD,*p < 0.05.

The ASP (at 60 cmH_2_O) was significantly higher in G-LMA than in P-LMA (*p* = 0.04) (Figure 3). The volume of air for cuff inflation to 60 cmH_2_O was significantly higher in G-LMA (22.60 ± 3.27 ml) than in P-LMA (13.90 ± 1.19 ml) (*p* < 0.0001).The device insertion time for G-LMA (13.20 ± 1.82 seconds) was less than that of P-LMA (15.95 ± 1.84 seconds) (*p* < 0.0001). The gastric tube insertion time for G-LMA (10.05 ± 1.35 seconds) was less than that of P-LMA (13.75 ± 1.74 seconds) (*p* < 0.0001).

We found no differences in successful insertion attempt (first attempt: G-LMA, 97.5%; P-LMA, 95%), ease of insertion (G-LMA, grade 1 = 30/40; P-LMA, grade 1 = 28/40), manipulation required to achieve effective airway, successful gastric tube insertion attempt (over all 100% within two attempt), ICP measurement, fiber-optic position of airway, blood staining (G-LMA, 20%; P-LMA, 22.5%) and postoperative pharyngolaryngeal morbidity (sore throat, dysphagia and dysphonia) at 1, 2, 24 hours after the surgery.

The parameters such as blood pressure, heart rate, end-tidal CO_2_, SpO_2_ were comparable between the two groups and were found within normal limit during the perioperative period. The hemodynamic and respiratory responses between the groups were found within normal limit in the post anesthetic care unit (PACU). Vomiting, regurgitation of gastric content, and minor trauma like lip/teeth/gums were not observed in both groups of patients.

## Discussion

This prospective randomized study was designed to compare the clinical performance of two laryngeal mask airways (P-LMA and G-LMA) and their postoperative pharyngolaryngeal morbidity. We found that the G-LMA had higher ASP with a short insertion time of the device as well as gastric tube as compared to P-LMA.

The ASP was measured as a primary outcome measure. It was measured when both the devices had air inflated to 60 cmH_2_O cuff pressure. We found that the mean ASP was higher for G-LMA than P-LMA. This may be due to differences in the shape and size of the cuff or single softer curved airway tube allowing full depth of insertion. High ASP indicates airway protection, feasibility of positive pressure ventilation and successful placement of LMA devices [8]. Verghese et al. [11] reported that at 60 cmH_2_O cuff pressure, ASP was identical while comparing PLMA and Supreme LMA (SLMA), in anesthetized female patients using neuromuscular blockade. Hosten et al. [12] demonstrated that in adult patients, SLMA had leak pressure similar to P-LMA. Seet et al. and Eschertzhuber et al. [13,14] reported that the ASP was higher for P-LMA than SLMA. Hosten et al. [15] claimed that the SLMA and P-LMA had similar leak pressure during laparoscopic cholecystectomy, suggesting G-LMA as a suitable airway device in laparoscopic cholecystectomy. Direct comparative study will be required to confirm this.

The cuffs of all devices were inflated to prefixed pressure (rather than a volume). To achieve 60 cmH_2_O, the P-LMA cuff requires larger volume of air than the classic LMA. This suggests that larger capacity of LMA device may increase seal pressure by allowing the cuff wall to match the shape of pharynx and larynx [16]. On the contrary, we obtained the volume of air which was significantly more in G-LMA than in the P-LMA. Instead of larger static cuff volume, the difference in volume required for obtaining target pressure was small. Small increases in the cuff volume caused marked increases in ICP [3]. This may be due to differences in the shape of the cuff and we assumed the cuff of G-LMA closely matches with the shape of pharynx and larynx, which might account for improved sealing pressure and low compliance during further inflation. We recommend a further study to confirm this.

The ICP was measured at 30, 60, 90 and 120 minutes and no difference was found between the groups. 60 cmH_2_O cuff pressure was maintained during surgery to eliminate the potential effect of pressure. As ICP of LMA devices increases, pharyngeal mucosal perfusion is progressively decreased, which results in postoperative pharyngolaryngeal adverse events [17]. Therefore, limiting cuff pressures of LMA devices can reduce postoperative pharyngolaryngeal adverse events [18-20]. Seet et al.[21] demonstrated that the use of manometry to limit ICP of LMA devices from 112 mmHg to < 44 mmHg (60 cmH_2_O) reduced pharyngolaryngeal adverse events in ambulatory surgical patients, but 60 cmH_2_O was still the higher limit in terms of airway perfusion pressure.We found no difference in postoperative pharyngolaryngeal morbidity (sore throat, dysphagia and dysphonia) at 1, 2 and 24 hours between two groups after the surgery. The cause might be associated with postoperative analgesic, which was not recorded in our study.

We found no differences in device insertion success, ease of insertion, gastric tube insertion success, and fiberoptic assessment of airway devices. We found that LMA insertion time was less in G-LMA than in P-LMA. As compared to P-LMA, G-LMA has single, softer curved airway tube that may be easy to insert. The drainage tube of P-LMA runs laterally with the ventilatory side of the airway device and later towards the tip of P-LMA and ends in the midline. However, the drainage tube of G-LMA is directly posterior to the ventilatory side and runs through the midline, and opens at the distal end of the cuff. We believe that an improved drainage tube design may explain the shortened insertion time of the gastric tube placement in G-LMA. The easy gastric access may be the additional safety benefit for the use of G-LMA to prevent aspiration.

The G-LMA has two extra features that are not present in the P-LMA: the hypopharyngeal port and visibly in-built ICP monitor. Formally, the efficacy of these features was not tested in our study but the ICP is roughly matched with the hand held pressure measuring device.

Our study has some limitations. We only used size-4 devices in adult patients with neuromuscular blockade; however, it can be presumed that same results would be obtained in patients who have not received neuromuscular blockade. Lastly, some of our data were not blinded, thus causing potential source of bias.

## Conclusion

We concluded that the G-LMA was associated with high airway sealing pressure during controlled ventilation and a quicker insertion of the device and gastric tube as compared to P-LMA in anesthetized adult patients who received neuromuscular drugs. The device insertion success, ease of insertion and postoperative pharyngolaryngeal morbidity were comparable. We recommend further study in multicenter, randomized trial with large number of patients.

## Additional file

Additional file 1:
**CONSORT 2010 Flow Diagram.**

## Abbreviation

ASP: Airway sealing pressure
G-LMA: Guardian laryngeal mask airway
ICP: Intracuff pressure
LMA: Laryngeal mask airway
PACU: Post Anaesthetic Care Unit
P-LMA: ProSeal laryngeal mask airway
SLMA: Supreme laryngeal mask airway
