# Supplementary material for: Comparisons of clinical performance of guardian laryngeal mask with laryngeal mask airway ProSeal
Source: BMC Anesthesiol. 2015 May 1;15:69. doi: 10.1186/s12871-015-0039-3 (PMC4429672; doi:10.1186/s12871-015-0039-3)
Supplement: Additional file 1: — CONSORT 2010 Flow Diagram. [file 12871_2015_39_MOESM1_ESM.doc]

**
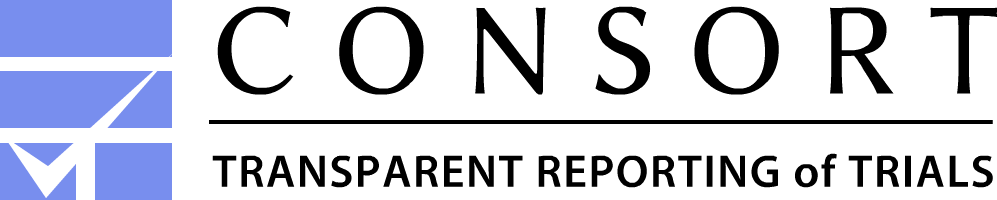
**

**CONSORT 2010 Flow Diagram**

**Allocation**

**Analysis**

**Follow-Up**

**Enrollment**

Assessed for eligibility (n=80)

Excluded (n=0 )

♦  Not meeting inclusion criteria (n=0 )

♦  Declined to participate (n=0 )

♦  Other reasons (n=0 )

Analysed (n=40)
♦ Excluded from analysis (give reasons) (n=0 )

Lost to follow-up (give reasons) (n=0)

Discontinued intervention (give reasons) (n=0)

Allocated to intervention (n=40)

♦ Received allocated intervention (n=40)

♦ Did not receive allocated intervention (give reasons) (n= )

Lost to follow-up (give reasons) (n= 0)

Discontinued intervention (give reasons) (n=0)

Allocated to intervention (n=40)

♦ Received allocated intervention (n=40)

♦ Did not receive allocated intervention (give reasons) (n=0 )

Analysed (n=40)
♦ Excluded from analysis (give reasons) (n=0 )

Randomized (n=80)
